# Blockchain Technology for Detecting Falsified and Substandard Drugs in Distribution: Pharmaceutical Supply Chain Intervention

**DOI:** 10.2196/10163

**Published:** 2018-09-13

**Authors:** Patrick Sylim, Fang Liu, Alvin Marcelo, Paul Fontelo

**Affiliations:** 1 National Library of Medicine National Institutes of Health Bethesda, MD United States; 2 Standards and Interoperability Lab for Asia University of the Philippines Manila Philippines

**Keywords:** supply and distribution, information systems, counterfeit drugs, blockchain

## Abstract

**Background:**

Drug counterfeiting is a global problem with significant risks to consumers and the general public. In the Philippines, 30% of inspected drug stores in 2003 were found with substandard/spurious/falsely-labeled/falsified/counterfeit drugs. The economic burden on the population drug expenditures and on governments is high. The Philippine Food and Drug Administration (FDA) encourages the public to check the certificates of product registration and report any instances of counterfeiting. The National Police of Philippines responds to such reports through a special task force. However, no literature on its impact on the distribution of such drugs were found. Blockchain technology is a cryptographic ledger that is allegedly immutable through repeated sequential hashing and fault-tolerant through a consensus algorithm. This project will develop and test a pharmacosurveillance blockchain system that will support information sharing along the official drug distribution network.

**Objective:**

This study aims to develop a pharmacosurveillance blockchain system and test its functions in a simulated network.

**Methods:**

We are developing a Distributed Application (DApp) that will run on smart contracts, employing Swarm as the Distributed File System (DFS). Two instances will be developed: one for Ethereum and another for Hyperledger Fabric. The proof-of-work (PoW) consensus algorithm of Ethereum will be modified into a delegated proof-of-stake (DPoS) or practical Byzantine fault tolerance (PBFT) consensus algorithm as it is scalable and fits the drug supply chain environment. The system will adopt the GS1 pedigree standard and will satisfy the data points in the data standardization guidelines from the US FDA. Simulations will use the following 5 nodes: for FDA, manufacturer, wholesaler, retailer, and the consumer portal.

**Results:**

Development is underway. The design of the system will place FDA in a supervisory data verification role, with each pedigree type–specific *data source* serving a primary data verification role. The supply chain process will be initiated by the manufacturer, with recursive verification for every transaction. It will allow consumers to scan a code printed on the receipt of their purchases to review the drug distribution history.

**Conclusions:**

Development and testing will be conducted in a simulated network, and thus, results may differ from actual practice. The project being proposed is disruptive; once tested, the team intends to engage the Philippine FDA to discuss implementation plans and formulate policies to facilitate adoption and sustainability.

**Registered Report Identifier:**

RR1-10.2196/10163

## Introduction

A recent report from the World Health Organization classified drug counterfeiting as a global problem. In low- to middle- income countries, an estimated 1 in 10 drugs in market circulation is falsified or substandard [1]. The consequences of this phenomenon pose significant risks to individuals and the public. They are most prevalent in areas where surveillance and regulation need improvement or are deficient and where medicines are in high demand but remain mostly unaffordable [2,3]. They are also rampant during disease outbreaks and epidemics when shortages of essential drugs tend to occur and when counterfeiting is most likely to rise.

Substandard drugs are dangerous. Falsified and substandard drugs, which could contain inactive ingredients, active ingredients but in the wrong dosage, or potential contaminants, could be lethal [4]. The lay press [5-8], replete with many personal anecdotes, as well as medical journals [9-12] have reported on the dangers of fake drugs. The use of antimicrobials of low quality may result in treatment failures and may increase antibiotic resistance in individuals and the community, resulting in higher mortality rates and the spread of highly resistant pathogens worldwide. Contaminants and impurities may induce allergic reactions and adverse drug reactions. Counterfeit drugs waste individual incomes and lead to increases in government economic burden. Furthermore, these may decrease the overall public confidence in the efficacy of authentic medicines [13,14].

The Philippine Food and Drug Administration (FDA), just like its US counterpart, has the mandate to ensure the safety, quality, and efficacy of food, medicines, and medical devices. The agency has repeatedly warned the public of fake pharmaceutical products peddled by counterfeiters that are circulating in the market. This warning comes with an advice to the general public to ensure that retailers where they obtain their drugs are certified by the FDA and that pharmacies display the Certificate of Product Registration, which the agency issues. In addition, the agency has a joint task force with the *Destroying Products Unfit for Human Consumption* (D-PUNCH) unit of the Philippine National Police [15]. The approach of D-PUNCH relies on the consumer reports of suspicious products or transactions to initiate action. In 2003, the agency reported that 30% of inspected drugstores were selling substandard/spurious/ falsely-labeled/falsified/counterfeit (SSFFC) drugs [16].

Drugs move across a distribution chain that involves several participants. These typically include, but are not limited to, a manufacturer, a wholesaler, and a retailer. A regulatory body, such as the FDA, may test the quality of a batch of drug product before or while it is distributed down the supply chain. These participants enter into direct contract-based relationships with each other: for instance, a retailer may enter a contract with a certain wholesaler to purchase stocks of a certain drug product regularly and another contract with another wholesaler to purchase stocks of a different drug product regularly.

Blockchain is an electronic cryptographic ledger that follows a decentralized network model—instead of storing all information in one database such as in conventional cloud-based applications, the information is distributed and synchronized across all nodes in the network. A consensus algorithm is deployed within the network to mitigate the issue of transaction duplication (or *double spending*) by allowing nodes to verify *true* information. Once verified, information is then added to the hash value of a previous *block*, and the new sequence (ie, previous hash + newly verified information) is hashed to form a new *block* using a cryptographic (ie, one-way) hash function. A cryptographic hash value is a string of nonreadable letters and numbers of consistent length that represent information that was subjected to a hash algorithm. Each hash value is unique to the information from which it was derived. These characteristics, in addition to the network forcing continuous synchrony across all nodes, make blockchain immutable and tamper resistant. Although cryptographic hashing is one-way, the decrypted information can be rehashed and compared with the stored hash value in the ledger. Furthermore, the network can persist amidst node failure. The threshold for the number of nonfunctional nodes before network failure is a function of the number of nodes connected to the network. The more the nodes in the network, the less likely it is to fail [17].

A review of current and emerging technologies to mitigate the incidence of fake drugs cited blockchain as an emerging technology, with the potential for tracking and tracing drug products and reagents, counterfeit detection through information verification of supply chain participants, and as an avenue for the integration of anticounterfeit devices into the internet-of-things and interoperability between unrelated databases in the supply chain [18]. It also has the potential for governance by enabling traceability, record ownership, incentivization through automation of smart contracts, and promotion of policy through multisectoral disruption [19].

In our review of literature, we found no previous studies or reports on the effectiveness or impact of consumer-driven Certificate of Product Registration checking and initiation of D-PUNCH investigations on mitigating the counterfeiting issue.

The applications of blockchain technology in clinical practice and health care research are currently of great interest and are being explored for the potential for increased security of health information amidst the increasing frequency of cyberattacks. Other domains outside health care are also exploring its potential in providing a trusted environment in which participants can provide and avail various services.

This study will test the feasibility of applying the technology and its principles in a pharmaceutical surveillance system and its resistance to tampering.

## Methods

### Pharmacosurveillance Blockchain System

#### System Design and Development

The system prototype will be a distributed application (DApp) with a back-end distributed file system (DFS) supporting a private blockchain network [20]. It will use smart contracts.

An instance will be developed on the Ethereum blockchain platform, which is open-source and currently one of the largest public blockchain networks, boasting an active community and a sizeable public repository of DApps. The platform currently uses a proof-of-work (PoW) consensus algorithm called Ethash; however, developers are planning to change it in the near future to a proof-of-stake (PoS) algorithm for scalability. Ideally, a delegated proof-of-stake (DPoS) or a practical Byzantine fault tolerance (PBFT) consensus algorithm fits the pharmaceutical supply chain environment, so modification may be necessary. Furthermore, Ethereum does not come with data encryption as default, which will incur additional development.

A second instance will be developed on the hyperledger fabric blockchain platform [21]. Unlike Ethereum, the platform is designed for private consortium networks. It is open-source, modularized, and uses data encryption and a DpoS or PBFT consensus algorithm by default.

Swarm, a DFS included as a native base layer service in Ethereum, is a good candidate for inclusion because of its default integration with the platform. The DFS component will store the DApp, smart contracts, and the blockchain. Swarm will be integrated into the system.

**Figure 1:**
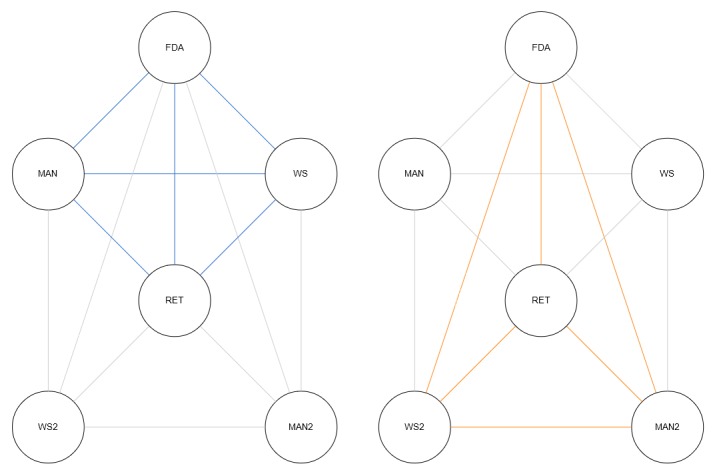
Two distinct distribution subchains within the blockchain network, highlighted blue and orange. The diagram on the left shows a distribution chain for a drug product, with blue lines representing distribution contracts. The diagram on the right shows a second distribution chain for another drug product within the same network, with orange lines representing distribution contracts. Client applications installed on nodes monitor transactions and track product movement. FDA: Food and Drug Administration; MAN: manufacturer; WS: wholesaler; RET: retailer.

The system prototype will be designed with 5 starting nodes, one for each participant in the traditional drug distribution model: the manufacturer, the wholesaler, the retailer, and the FDA, as well as an additional node that will house a consumer portal website through which consumers can scan codes that come with the receipt of their purchases to view the drug distribution history. Smart contracts will be used to define contract-based relationships between participants, and the interfaces will reflect supply chains in which the logged account is involved. While data will be distributed across the DFS, accounts will only be able to visualize and decrypt files intended for them—in other words, subchains will exist within the network as shown in Figure 1.

Despite the orientation of the internode connections within the network, the movement of a drug product along the distribution network will be distributed across all nodes. The DApp would have the capacity to detect anomalies, unauthorized data insertions, and missing drug products by comparing DFS content with ledger records. Each step will be tagged with a timestamp for auditing.

The DApp front end will be stored in all nodes. Its interface will include a section that will display transactions performed along the distribution chains as well as detect anomalies and information discrepancies in a dashboard. It will trace the drug product as it moves along the chain and generate a timeline for each supply chain. Notifications will be displayed for shipments and anomalies detected in the chain. It will also allow the FDA account to define authorized manufacturers and dealers and store the definitions in a smart contract or encrypted file.

**Figure 2:**
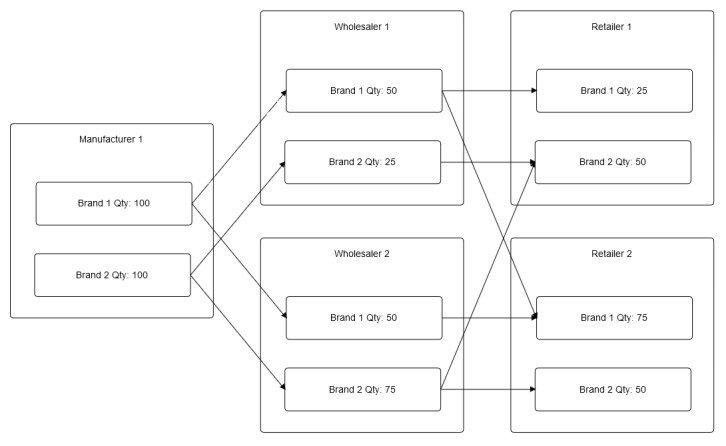
Possible branching and merging patterns in the drug distribution chain.

The system will adopt the GS1 pedigree standard [22] recommended in the Drug Supply Chain Security Act. The DApp will allow the drug supply chain participants to create product manufacture pedigrees, shipping pedigrees, and receipt pedigrees (where and when applicable), with each pedigree electronically signed and appended to the overall pedigree by each *author* (participant) down the supply chain. The content of each pedigree will satisfy, at minimum, the Standardization of Data and Documentation Practices for Product Tracing Guidance for Industry document published by the FDA [23]. The global identifier number (GTIN) will be used for identifiers.

##### Food and Drug Administration Account

The FDA account will have access to functions that allow the user to add information to reference smart contracts that define authentic drug products, supply chain participants, and contract relationships. Information uploaded by this account to the network will be considered authentic and will be used as the reference against which documents in the DFS will be checked. All sections and functions of the client application will be accessible to this account. Furthermore, this account verifies all transactions—all other accounts will automatically publish a session key encrypted with the FDA public key when they attempt to upload a file into the DFS.

##### Manufacturer Account

Information uploaded by this account to the network will have credentials and certificates linked and will initiate supply chains, which the system will subsequently track using the pedigree files in the DFS. Upon verification of identity and registered distribution contracts linked to the specific brand of drug product, the system will determine whether the merchandise moves along a registered chain and will verify consistency of information through each node.

##### Wholesaler and Retailer Accounts

Information uploaded by these accounts to the network will have credentials and certificates linked and will be validated by the system against the registries.

Distribution chains may branch out or merge at certain nodes, and the system should detect such patterns when it visualizes them into timelines. For improved auditing, the system will also track the amount or stock number of each brand of drug product that moves across each node, using the various pedigrees submitted by the supply chain participants.

Branching and merging patterns in Figure 2 will be visualized into 6 separate timelines, namely the following:

1. Brand 1: Manufacturer 1 (100), Wholesaler 1 (50), and Retailer 1 (25)
2. Brand 1: Manufacturer 1 (100), Wholesaler 1 (50), and Retailer 2 (25)
3. Brand 1: Manufacturer 1 (100), Wholesaler 2 (50), and Retailer 2 (50)
4. Brand 2: Manufacturer 1 (100), Wholesaler 1 (25), and Retailer 1 (25)
5. Brand 2: Manufacturer 1 (100), Wholesaler 2 (75), and Retailer 1 (25)
6. Brand 2: Manufacturer 1 (100), Wholesaler 2 (75), and Retailer 2 (50)

The system interface will include a mechanism by which drug products can be repackaged and shipped. Packages will be represented by receipt and shipping *pedigree envelopes* and will contain the GTINs of all drug products included in the package.

**Figure 3:**
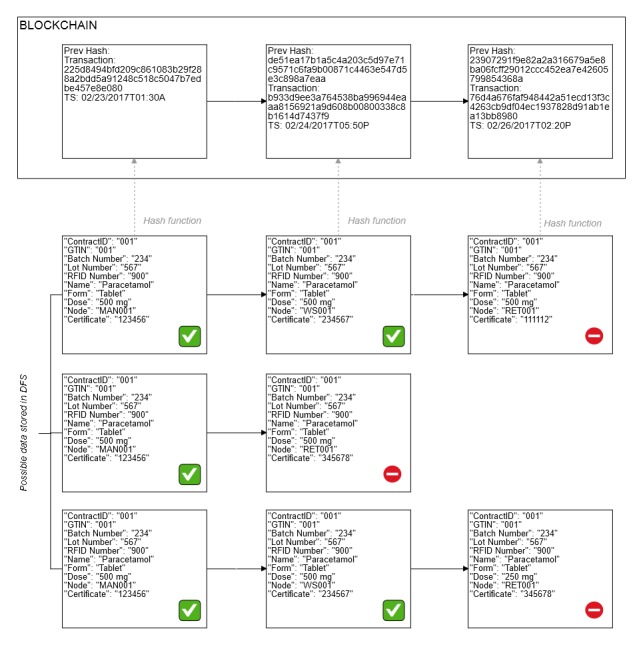
Example of anomalies that can be detected. Top row, information added by a node contains the wrong certificate. Middle row, information is missing from the block. Bottom row, drug product information is different from previous records or does not match the record in the distribution contract registry.

Figure 3 shows an example of data in the DFS and blockchain ledger records. When a manufacturer ships a batch of a drug product, a shipping pedigree is submitted into the network and the record is verified and counterchecked against the blockchain ledger by the DApp account of the recipient by using the Keccak-256 cryptographic hash function (used in Ethereum). It will send a notification to the network if the hash values do not match. In Figure 3, those marked with anomalous content have hash values different from those recorded in the blockchain ledger. The examples of anomalous information notifiable events are when certificates linked to the node identification do not match with those on record (hinting fraud), information supposedly recorded by a node is missing from the block (hinting at node failure or a skipped participant), and when the hash value of specific drug product information does not match its counterpart on record (hinting a typographical error or tampering of the drug product). All scenarios would need action from the FDA (or the regulating agency).

### Database

The system database will be a DFS with 4 main parts:

1. The blockchain ledger.
2. The smart contracts repository, where real-world contracts, participants, and drug products will be defined.
3. The document repository, where the pedigrees will be stored.
4. The drug distribution history that contains a listing of the participants who possessed the drug at some point during the distribution, as well as information on shipments.

### Radio-Frequency Identification Tags

Radio-frequency identification (RFID) tags will be used as an additional data point linked to a particular drug product. Each node will have an RFID scanner. RFIDs will be incorporated to drug product packages at the manufacturer level and will be added as a data point in pedigrees down the supply chain. Data mismatches will trigger notifications.

### Drug Product Pathway

The following describes the proposed pathway for a drug product along the drug supply chain and how the proposed system will integrate into the workflow:

First, the process is initiated when a manufacturer creates a drug product and assigns a GTIN to each physical unit of drug product. A manufacturing pedigree is created for each physical unit of drug product and entered into the system. This document is then encrypted with a generated session key, which is then encrypted with the private key as well as the public key of the FDA account and stored in the DFS. The document is linked to its hash value in the DFS. The hash value is submitted to the network for verification. The FDA account is pinged by a premade smart contract and verifies each manufacturing pedigree by checking the credentials of the manufacturer.

Second, the manufacturer then groups the drug products into a shipment package and assigns an RFID. A shipping pedigree is created for each shipment package and entered into the system. Hash values of manufacturing pedigrees of drug products included in the shipment package are listed in the shipping pedigree. The session keys used previously are encrypted with the recipient public key and stored in the shipping pedigree. The shipping pedigree is then encrypted with a generated session key, which is then encrypted with the private key as well as the public keys of the FDA account and the recipient account. The shipping pedigree is identified by its hash value in the DFS. The hash value is submitted to the network for verification. The FDA and recipient accounts are pinged by premade smart contracts and verify each shipping pedigree by checking the credentials of the manufacturer and that the manufacturing pedigrees referenced in the shipping pedigree exist in the DFS and have been previously verified.

Third, the distributor receives the physical shipment package from the manufacturer and scans the RFID. The system then verifies that the RFID matches a shipping pedigree received in the DApp inbox and opens the pedigree. The recipient verifies that the manufacturing pedigrees referenced in the shipping pedigree exist and the GTINs match those of the physical drug products. The recipient then creates a receiving pedigree with the hash values of the manufacturing pedigrees of drug products actually received. This document is then encrypted with a generated session key that is then encrypted with the private key as well as the FDA’s public key. The document is linked to its hash value in the DFS. The hash value is submitted to the network for verification. The FDA account verifies the pedigree like above.

Finally, a recursive process of shipment, verification, receipt, and verification occurs until the point of sale. The consumer receives a code along with the drug products purchased. The consumer then scans the code with a mobile phone camera, and is directed to a consumer portal where the distribution history of the drugs in the receipt is displayed.

### Anomaly Detection

The system will be designed to detect 5 types of anomalies anywhere along the chain:

1. Missing nodes in the distribution chain.
2. Distribution chains that have not completed after a certain threshold.
3. Invalid node certificates.
4. Unregistered products entering the distribution chain.
5. Primary data point (ie, drug-related data, eg, dose) discrepancies.
6. Timestamp anomalies.

### System Testing

After the development of the prototype, testing will be performed in a simulated network environment, evaluating 2 main parameters:

1. Benchmarks on various data corpus sizes (as a function of the number of transactions).
2. Capacity of the system to reliably detect the anomalies described above.

Analyses will be performed on the feasibility of the system for large-scale implementation based on these 2 main parameters. Assumptions and recommendations will be discussed in their respective sections.

## Results

### Interfaces

The system will have a DApp with a front end that provides a user interface. The DApp will have permissioned access to documents and records despite the said files existing in all nodes.

### Log-In

**Figure 4:**
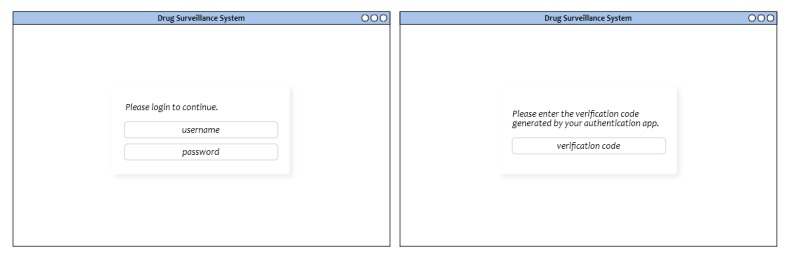
Log-in interface with two-factor authentication.

The application will be locked behind a log-in interface with two-factor authentication as shown in Figure 4. It will ask for user credentials (username and password), as well as a verification code sent through an authenticator application. All credentials, including those for the authenticator application, will be issued by the FDA through the user management module integrated into the system. There may be several users for each supply chain participant account, and while each participant account is issued one pair of keys, the pedigrees submitted to the network will contain information on the currently logged-in user.

**Figure 5:**
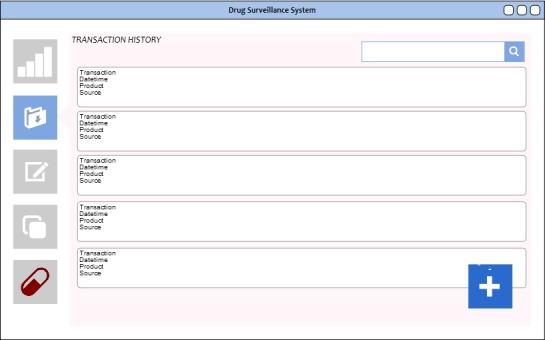
Transaction history, listing transactions made by the participant using the node.

### Transaction History

The transaction history shown in Figure 5 is a simple data visualization widget that will be accessible to all accounts. Although the design involves the distribution of pedigree files across all nodes connected to the network, each file will have defined permissioned *recipients*, encrypted with session keys that would be, in turn, encrypted with keys of intended recipients. The (+) button at the lower right portion of the screen allows the participant to create a pedigree, append it to the overall document, and submit it to the network for verification.

### Timeline Dashboard

**Figure 6:**
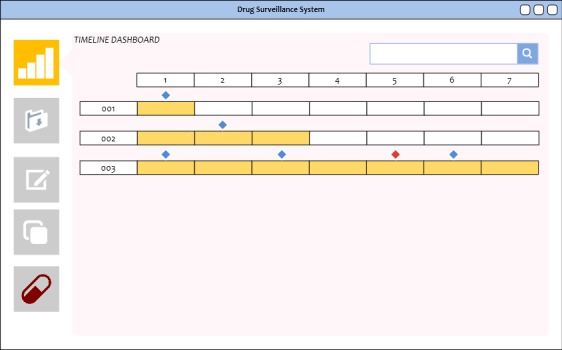
The timeline dashboard to visualize transactions along a distribution chain to highlight manufacturer shipment time to consumer purchase at a retailer. Diamond markers signifying transactions. A red diamond denotes a possible problem based on the information distributed on the network.

The timeline dashboard generates graphs that illustrate the progress of a drug product along the supply chain. Anomalies in the information recorded by nodes will be marked with a red badge as shown in Figure 6. These badges can be clicked to show the latest submitted information from that particular node. The DApp will automatically verify pedigree documents in the DFS with the records in the blockchain ledger, the manufacturing pedigree, and the defined supply chain smart contracts. Should a malicious party insert a fraudulent document into the repository, bypassing an intended recipient, the system will be able to detect the anomaly.

### Contract Registry

**Figure 7:**
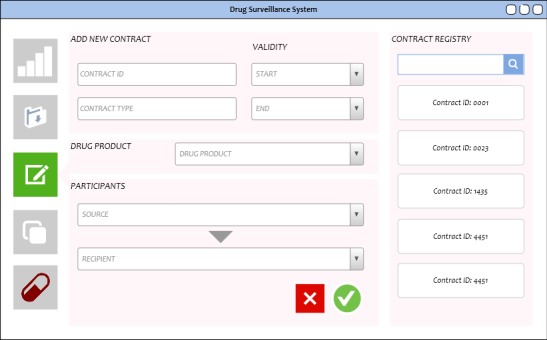
Contract registry interface only installed on the Food and Drug Administration (FDA) node and accessible to the FDA account.

Figure 7 shows the screen after the *Contract* button (pen and paper) on the left panel is clicked and a new contract form is opened. Hovering over the contract icons to the right will display a small window with the details for that contract.

**Figure 8:**
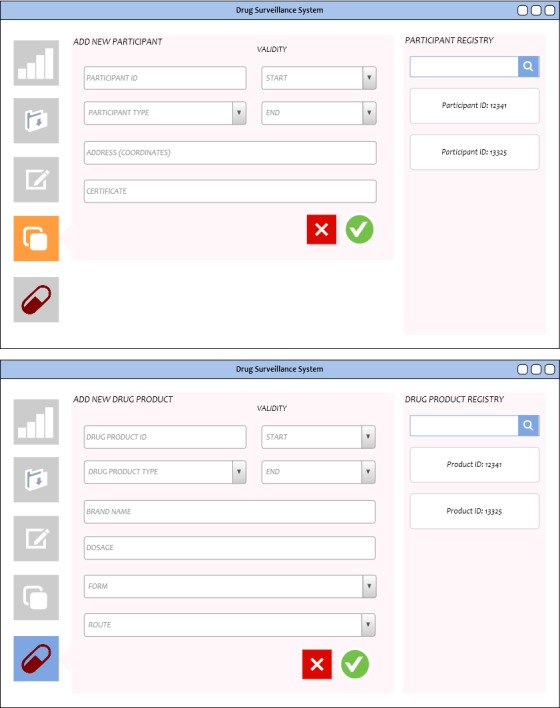
Participant and drug product registries. The corresponding forms for adding participants and drug products into the network are shown. These interfaces will only be accessible by the Food and Drug Administration.

The smart contract repository will contain the canonical definitions through which the system can detect information anomalies and broken supply chain sequence of events. The interface will refer to the set of definitions in the repository as *registries*. Only the FDA account can add and edit contracts. In the blockchain, once a contract is committed, the record of its creation cannot be modified, tampered with, or deleted.

A contract will minimally contain the name of the drug product, a source and recipient (both should be FDA-certified and registered to the system before the creation of the contract), and other metadata such as unique identifiers, certificates, and start and end dates.

By design, there will be one contract assigned to each drug product transaction between 2 supply chain participants. At any point in time, a participant may have several contracts with other participants. The definitions will be instrumental in verifying transactions in the supply chain by allowing 2 layers of verification: first, the sender should be legitimate and certified by the FDA, and second, the hash values of the data in the DFS and the blockchain ledger must match, and the product information in the DFS must match those in the definitions.

### Participant Registry and Drug Product Registry

The participant and drug registries serve to feed the input fields of the contract registry, containing the necessary data to distinguish between participants as well as between drug products (Figure 8). As with the contract registry interface, these will only be accessible by the FDA account. An option would be to allow other accounts to view the registries for reference; however, adding and editing privileges will be given only to the regulating agency.

## Discussion

### Technology Adoption

The pharmacosurveillance blockchain system being proposed is a highly disruptive intervention, particularly in the context of drug supply chains in a low-resource country such as the Philippines. It is projected to affect not just the pharmaceutical industry but the entire distribution chain and the consumer. Adoption and sustainability can only be achieved with consumer awareness and empowerment, as well as sound policy backing and good governance. The assessment of adoption potential at the feature level using the unified theory of acceptance and use of technology (UTAUT) and its extensions [24] for each affected sector may be performed once the technology has been developed and tested. In UTAUT, system performance, effort expected, social influences, and prevailing conditions will determine whether the introduction of new technology or device will be successful. In a developing country such as the Philippines, the last 3 components are likely to be the least predictable in determining the success of this disruptive technology.

As a portion of distributed SSFFCs travel outside the official drug supply chains, the system cannot detect them until they have reached the consumer. This is where consumer awareness and empowerment will play a significant role in the implementation: first, consumers should be aware that their purchases should come with a receipt that has a distribution history code that they can scan to verify the authenticity of the drug, and second, they should be empowered to report discrepancies to the FDA. In turn, the FDA has to have the capacity to accommodate, process, and respond to reports from the system and consumers.

In terms of policy, we recognize two important potential issues to resolve for successful adoption: first, local and national laws will have to recognize blockchain ledger records as a *source of truth*, admissible as evidence in the court of law. Second, policy will have to incentivize investments in infrastructure and human resources on the part of the participants in the drug supply chains. The FDA or another government agency will take on a capacity-building role, training key personnel from participants in the drug supply chain, not only on the use of the system but also the principles and best practices that facilitate the mitigation of the distribution of SSFFCs.

A DpoS or PBFT consensus algorithm is ideal for the project for several reasons: first, it eliminates the need for third-party *miners*, who would compete for computing power under PoW and currency under PoS, in a setting where resources are low and in an industry where participants have equal stake in the success of their own supply chains. Second, it is economical in terms of power consumption. Third, it is better designed for private consortium networks, of which the proposed system is an example. The FDA will be central to the verification process, typically paired with another participant with the authority to verify contract-specific information, for instance, a wholesaler must verify that the drugs in a recently received package and the shipping and manufacturing pedigrees match.

### Assumptions and Limitations

Aside from assumptions on resources and infrastructure, the design of the prototype makes several additional assumptions that may affect implementation: first, that there is a regulatory agency such as FDA that exists and is monitoring the drug marketspace. Second, that the regulatory agency has the practice of certifying manufacturers, wholesalers, retailers, drug products, and reagents and has the practice or capacity to keep records of the certifications. Finally, that the participants in the drug supply chains implement, or have the capacity to implement, standards on the metadata surrounding the drug products with which they conduct business. Another major assumption is that the participants in the pharmaceutical distribution chain— manufacturers, distributors, retailers, and finally consumers are willing to participate in this disruption in the usual conduct of commerce. The consumer in the end will be willing to verify the authenticity of drug products and report anomalies that may be found. This will require awareness, training, and the desire to create an environment of authentic medication in the supply chain. In the event that these conditions are not met, a strategy to consider is to approach and engage the proper authorities: for instance, a government agency that regulates the drug product and reagent marketspace in the region, or certain policy makers and leaders with a pharmaceutical or public health focus that can formulate and push legislation to facilitate changing the environment favorably. In addition, a strategy to consider is to use the findings from the UTAUT analysis.

The study design has the following limitations:

1. The proposed system will only be able to detect drug movements that follow official distribution chains known to the regulatory agency. It cannot track falsified drugs that are distributed through routes outside of official distribution chains.
2. The proposed system will be developed and tested in a controlled simulated network; therefore, results obtained from this study may not be reflective of actual performance when deployed in a real-world setting.

## Abbreviations

DApp: distributed application
DFS: distributed file system
DPoS: delegated proof-of-stake
D-PUNCH: Destroying Products Unfit for Human Consumption
FDA: Food and Drug Administration
GTIN: global identifier number
PBFT: practical Byzantine fault tolerance
PoS: proof-of-stake
PoW: proof-of-work
RFID: radio-frequency identification
SSFFC: substandard/falsely-labeled/falsified/counterfeit
UTAUT: unified theory of acceptance and use of technology
